# A supervised visual model for finding regions of interest in basal cell carcinoma images

**DOI:** 10.1186/1746-1596-6-26

**Published:** 2011-03-29

**Authors:** Ricardo Gutiérrez, Francisco Gómez, Lucía Roa-Peña, Eduardo Romero

**Affiliations:** 1 Telemedicine Centre, National University of Colombia, Carrera 30 No. 45-03, Medicine Faculty, Building 471, Bogotá, Colombia

## Abstract

This paper introduces a supervised learning method for finding diagnostic regions of interest in histopathological images. The method is based on the cognitive process of visual selection of relevant regions that arises during a pathologist's image examination. The proposed strategy emulates the interaction of the visual cortex areas *V*1, *V*2 and *V*4, being the *V*1 cortex responsible for assigning local levels of relevance to visual inputs while the *V*2 cortex gathers together these small regions according to some weights modulated by the *V*4 cortex, which stores some learned rules. This novel strategy can be considered as a complex mix of "bottom-up" and "top-down" mechanisms, integrated by calculating a unique index inside each region. The method was evaluated on a set of 338 images in which an expert pathologist had drawn the Regions of Interest. The proposed method outperforms two state-of-the-art methods devised to determine Regions of Interest (RoIs) in natural images. The quality gain with respect to an adaptated Itti's model which found RoIs was 3.6 *dB *in average, while with respect to the Achanta's proposal was 4.9 *dB*.

## Background

A typical pathology laboratory examines more than 100 microscopical slides per day [1], a scenario in which its workflow is based on the interaction of the pathologists with a conventional microscope. Digitization brings several advantages over the physical slides at facilitating communication between specialists, annotation of relevant structures and interaction between pathologists and virtual slides [2]. However, the lack of standardized criteria to preserve data reliability -from the early capturing process to the final interpretation-, limits the routine used of virtual microscopy techniques, in despite of its obvious technical advantages, namely, second opinions, team work, image annotation, deterioration-free digital storing. Such a standard should provide a robust frame, allowing the pathologists to achieve proper diagnoses, since it should also garantee that the image data will be free of any artifact introduced during the slide preparation, digitization, transmission or visualization. This standard should deal with three main questions: 1) What quality level meets the minimal diagnosis conditions, avoiding wrong diagnosis decisions? (legal aspect), 2) What quality level is needed for accurate diagnoses? (medical aspect) and, 3) How to measure the image quality for diagnosis? Which is the maximum quality level given by an automated process? (technical aspect) [3]. Moreover, in terms of the diagnosis quality, it is well known that different types of slides require different level of quality, i.e., simple and routine slides require lower quality levels than complex and rarely ones [4]. Furthermore, image regions, considered as relevant, require in general higher quality levels.

A reliable determination of clinically meaningful Regions of Interest (RoIs) in medical images is at the very base of strategies for selective image analysis, adaptive delivering of image data and clever compression algorithms. A proper determination of these RoIs would allow to concentrate any processing effort on specific image areas, relevant within a particular context. This fundamental statement would improve the processing performance in applications such as medical education, medical training, decision support systems, virtual microscopy and telepathology, among others [5-7]. The RoI analysis would allow to efficiently cope with large quantities of data, a crucial issue in many medical specialities [5,8,9]. For instance, a 1 *cm*^2 ^digitization of a physical slide at a level of × 20 magnification, results in a microscopical virtual slide of about 4 *GB *[10], a real time challenge even for modern communication networks. In the pathology literature, there exist several studies that have shown that not all information in a slide is relevant [11,12]. Expert pathologists draw their attention to some particular structures when exploring a microscopical slide [12]. Different approaches have attempted to find these RoIs with any level of repeatability, but so far the typical one has been to manually draw these regions [11]. This method is, obviously, time consuming and presents high inter-observer variability, in some studies up to a 20% [13]. Automatic RoI extraction in histopathological images is a very challenging task because of their very complex color, shape and architectural variabilities [14]. This picture is even worst if one thinks that histological samples are randomly taken from a lesion and that the anatomical biopsy is cut at different orientations and locations [15]. The naive use of current low level-RoI-extraction methods for medical images would probably fail since they disregard main histopathological issues such as particular the tissue architecture and the relations between different structures [16,17]. An expert nevertheless is capable to weight each of them and figure out a very precise diagnosis. Attention is herein thought of as the system capacity to select relevant information in function of a particular task. Some computational attention models rely on low-level image features to locate the relevant or conspicuous information within an image. One of these "bottom-up" models of attention, is the one proposed by Itti et al. [18]. Other theoretical and computational models of attention rely on "top-down" information, i.e., memory (semantic, episodic, declarative) and specific behavioral tasks [19].

The main contribution of this work is to model the visual process of recognition by bringing together the effect of the simpler cells of the visual cortex V1 (bottom-up information) and its interaction with more complex structures: V2 and V4 cortexes (top-down information). Through these interactions, this model emulates the pathologist's first examination step where she/he defines and separates high informative diagnostic regions [20]. Thus, the idea is to capture groupings, not necessarily neighbors, endowed with similar histopathological meaning. The method combines the advantages of a low level image characterization with a high discriminant power in terms of tissular properties and spatial grouping, information learned from the pathologists. This novel strategy was assessed in basal cell carcinoma images stained with Hematoxylin-Eosin, but is extensible to other histopathological images since the methodological analysis is alike in many other medical entities. This carcinoma is a representative tumorous pathology constituted of abnormal epithelial and connective tissue arrangements, which are also found in many other pathologies [15]. Our results demonstrated more similar RoIs to the pathologist's selections than those obtained with two classic strategies of visual attention.

This article is organized as follows: the problem and some previous works are introduced in this section, Section Materials and Methods is devoted to describe the proposed method for finding relevant information regions, evaluation and experimental results are presented in Section Results and some conclusions and perspectives are discussed in the last Section.

### Related Work

The problem of selecting RoIs has been approached in several medical image modalities. For instance Karras et al. [16], using gray scale pictures from abdominal cancer, assumed that regions with high density of repetitive patterns were more relevant than others. A robust description was obtained by using a vector of texture characteristics like energy, correlation, inverse difference moments and entropy. These features were the input to a fuzzy c-means clustering algorithm that classified regions as important or non-important. Gokturk et al. [8] claimed that relevant information in CT colon images was mainly due to the boundaries, when they are separated by air from other tissues and are recognized as variations on the gray scale levels. This kind of strategies could not be straightforwardly applied to histopathology images because these techniques ignore information such as color, intensity or spatial correlation [17,21], crucial in these images since they are basically characterized by a repetitive complex mix of these patterns. A classical approach, in natural images, has consisted in finding RoIs with high spatial edge density [22]. Again, this concept could hardly be applied to histopathological images because they contain regions with high edge concentration without clinical meaning [23] so that this approach would surely fail.

In the histopathological domain, a similar problem has been previously approached in automatic cancer diagnosis, case for which the aim was to automatically decide on the existence of cancer by examining the tissue properties [24]. These properties were characterized at two levels: cellular, focusing on cell abnormalities, [25,26] and tissular, by description of changes in cell distributions [27]. The analysis in both cases was performed by low level image characterization and a statistical analysis to discriminate normal from cancerous tissues. A large variety of low level image features has been used in histopathology: morphological, textural, fractal, topological and intensity based features [24]. These features are always computed at the pixel level, regardless the fundamental fact that histopathological images are constituted by objects [20]. A recent work in this direction was proposed by Tosun et al. [14]. In colon biopsy images, they approached the histopathological objects by circular primitives, upon which they computed an homogeneity measure. A growing and merging algorithm was used to segment cancerous tissues by minimizing these measures. Unfortunately, these algorithms highly depend on many non-intuitive parameters [14,27], which must be manually tuned.

A pathologic diagnosis is the result of a complex series of activities mastered by the pathologist. Classical psychophysical theories suggest that complex visual tasks, such as histopathology examination, involve high degrees of visual attention [20]. There exists evidence showing that focal attention, displayed serially to different locations, integrates the constituting low level features of an object [28]. These findings have inspired several computational algorithms that somehow search to structure the low level features [29]. One of the most influential is the one proposed by Itti et al. [18], a pure bottom-up attention model that locates relevant foci, based on a conjoint map of three low level characteristics: color, intensity and orientation. Although this method has been successfully tested in natural images, primary results on histopathological ones were not (as it would be described later). The relevant semantic information of these images is mainly constituted by repetitive patterns, which cannot be linearly reconstructed from the three basic features used in Itti's model. As far as we know, the unique visual model has been proposed by Achanta et al. [30], aiming at identifying regions for which the level of attention is as uniform as possible under the restriction that the region must conserve edges. A well defined object is defined at segmenting the original image with a mean-shift clustering algorithm, on which the saliency mean is computed within the resulting areas. These computational models have been used to characterize RoIs in natural images [31], but their use in medical images has remained very limited.

## Materials and methods

### Images and Ground Truth

**Figure 1:**
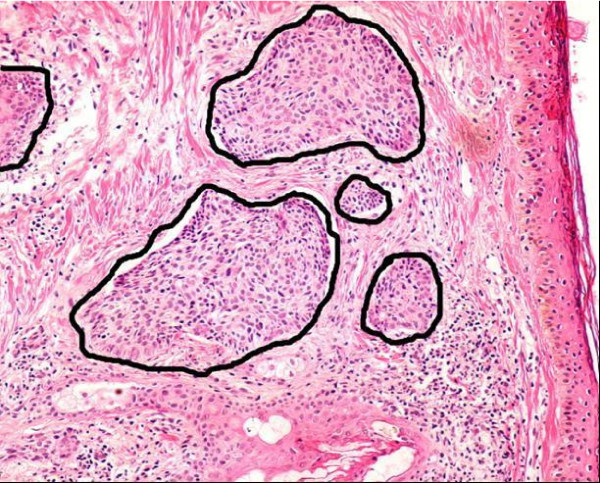
**Ground Truth**. Illustration of a ground truth, drawn by an expert pathologist.

A total of 338 histological microscopical fields of view of different types of basal cell carcinoma, sampled from 25 randomly chosen patients, were selected for this evaluation. The set of evaluation was composed of microscopical fields taken at different objective magnifications, namely, 37 were captured using a × 4, 148 using a × 10, 83 using a × 20 and finally 70 using a × 40 magnification. Each biopsy was formalin-fixed and stained with Hematoxylin-Eosin dyes. Microscopical fields were digitized with a Nikon eclipse E600 system, through a coupled Nikon DXM1200 camera, and stored in JPEG format at a 1280 × 1024 resolution. An expert pathologist, with at least five years of experience, selected the digitized fields of view and manually segmented relevant regions. One of these manual segmentations is shown in Figure 1.

Notice how difficult is to define a border, the tissue inside an islet is more cluttered than the outside, and the carcinoma is highlighted by Haematoxylin-eosin. It should be strengthen out that in this kind of images, the color is very alike so that this characteristic has a low discriminative value.

### Method Overview

A routine pathologist's visual examination is carried out in two sequential phases, when exploring a microscopic slide. An initial search for coarse tissue structures at a "low zoom" [20,32] to separate the image into large regions, and a subsequent finer feature characterization at a "higher zoom" within these regions to identify cellular structures [33]. On the other hand, other authors have found that pathologists analyze two main information sources within a slide: 1) Information associated to biological structures, looking for abnormalities such as atypical nuclei sizes, external material or structural disorders, and 2) Non-objectual information, mainly related to information about the type of tissue or disorder. Somehow, a combination of these sources, leads to a precise diagnosis [4,34].

The approach proposed herein attempts to emulate the pathologist's initial examination step where she/he defines the different regions of the image according to the inherent properties of each tissue type, such as level of visual attention and texture. Our approach tries to identify which of these regions are of diagnostic interest in a similar way as a pathologist decides where to look for finer details. The idea is to capture groupings, not necessarily neighbors, endowed with histopathological diagnostic meaning. These groups are determined by the similarity relationships between the objects inside them. The activation degree within each group is regulated by specific characteristics learned from the task. Finally the groups compete among them to win the pathologist attention.

**Figure 2:**
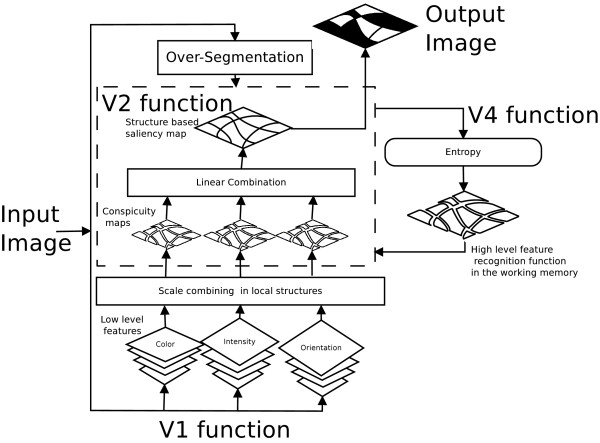
**The proposed method diagram**. The proposed method finds structures with diagnostic meaning and assigns a level of relevance to each one. At the first step, our method split the image into several structures. After that, the low level features compete locally to attract the user attention. Then the high level features regulate the activation map. Finally it identifies the relevant regions in the image.

The proposed strategy (Figure 2) emulates the interaction of the visual cortex areas *V*1, *V*2 and *V*4 [35,36]. Our model, based on this type of associations, integrates these three stages as follows: 1) Using the conventional Itti's model, local conspicuity regions are set, using exclusively low level features. 2) The conspicuity maps, coming from the precedent phase and an oversegmentation, are the input to this stage. The oversegmentation algorithm minimizes the within-class variance whose parameters are learned from the problem. The *V*2 function integrates the low level features with the oversegmentation by averaging the local conspicuity into each region. 3) The saliency map is computed using two types of information: a measure of the texture pattern (simulates the *V*4 → *V*2 interaction) and the previously described conspicuity maps (*V*1 → *V*2 interaction).

As a first approximation to this problem, there is no reason to prefer texture or low level features. Therefore, we linearly combine them and an independent threshold on each of the segmented regions was defined as the calculated saliency value which was larger than the saliencies regions mean.

### Grouping structures in histopathological images

**Figure 3:**
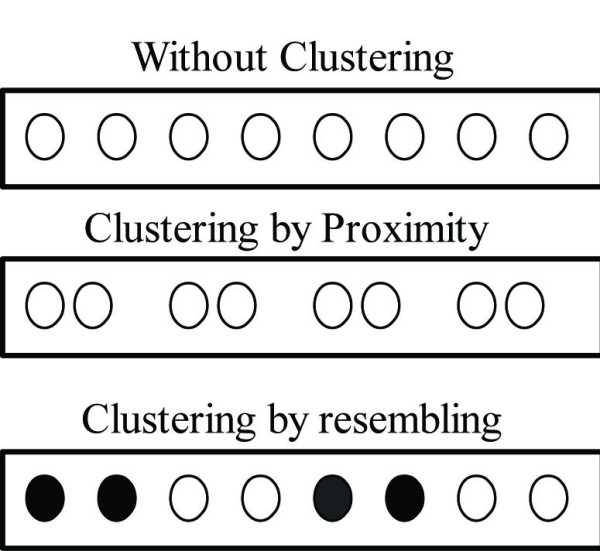
**Gestalt law**. Gestalt laws for proximity and resembling.

Visual attention is the ability of a biological or artificial system to find relevant region in a scene [29]. In the particular case of humans, they can not only find relevant regions, but also recognize complex structures in a scene. The Gestalt laws for proximity and resembling, illustrated in Figure 3 have motivated the fundamental hypothesis of our model, i.e., a histological tissue is a grouping of objects which resemble in their very basic structural properties.

Visual systems reach grouping by clustering, proximity and resembling. Any strategy should at least look for any of these basic properties, just like carcinoma stained images have cluttered regions composed of simple structures with similar average intensity. The grouping characteristics defined before are herein used to segment, based on a comparison of the intraclass and interclass variances regarding the intensity value of each pixel. This strategy was implemented using the Felzenszwalb algorithm [37] as follows: 1) pixels are sorted out by similar intensity value, and 2) neighborhoods are organized by grouping pixels whose intensity values were defined under a variance threshold. This method provides a still-segmentation strategy which is inspired from psychological grouping theories [38]. The main idea is that two regions are perceived as different if differences between them are larger than differences within them, according to a learned rule. The problem is defined in terms of a graph, where a non-linear decision function specifies if two elements *c*_1_, *c*_2 _in a graph partition should merge or not. The decision function reads as:(1)

The two regions *c*_1 _and *c*_2 _are merged together when *M *(*c*_1_, *c*_2_) is one,  depends on the size of *c *(|*c*|) and establishes an evidence for a boundary between two components, *k *is a scaling factor that sets preferences for specific component sizes, Diff*_wR_*(*c*) corresponds to a within-region difference which stands for the largest difference inside the component, while Diff*_bR_*(*c*_1_, *c*_2_) is a between-region difference that looks for evidence of a boundary between both components [37].

### Automatic Still-Segmentation of Histopathology Images

**Figure 4:**
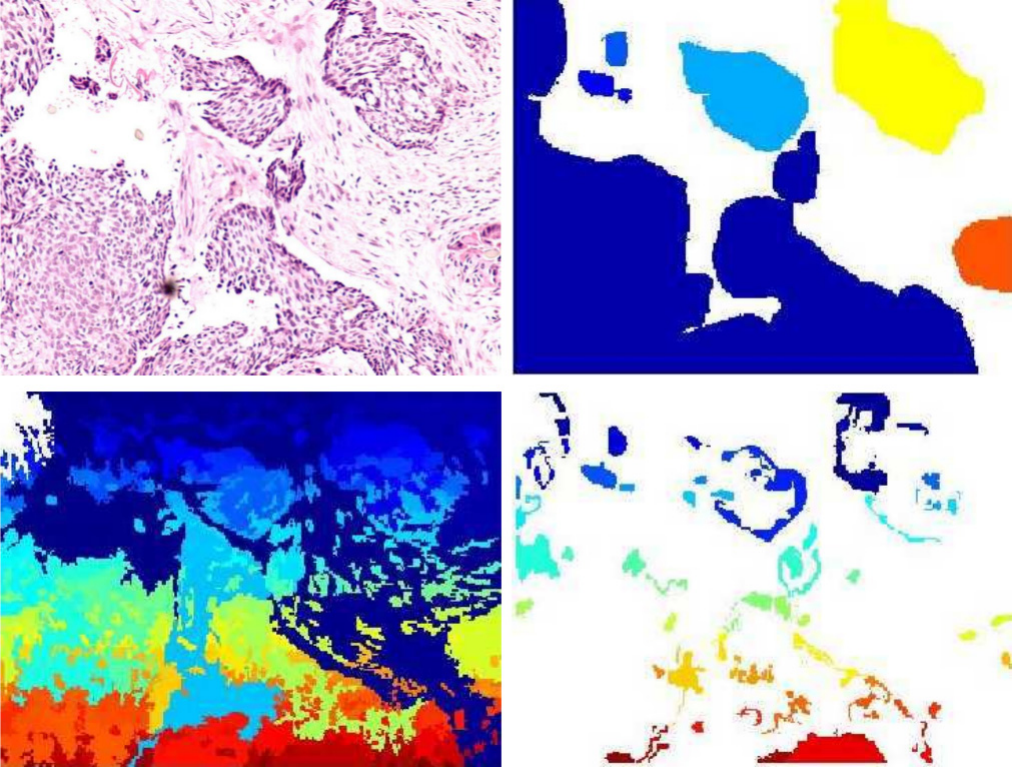
**Segmentation parameter selection**. Results of a wrong selection of the segmentation parameters. Panel at top-left shows the original image. At the top-right, the RoI manually segmented by the pathologists. At the bottom-left, a result of oversegmentation and finally, at the bottom-right, a result of under-segmentation.

The previous algorithm can be used to split the histopathology image into its constitutive tissue parts. As observed in Figure 4 the quality partition is highly dependent on the segmentation parameters.

A manual selection of these parameters is always possible, but this is by no means an intuitive task for the expert. Therefore, we proposed an energy-based learning method for selecting an optimal set of segmentation parameters, based on manually segmented images. Let *I *a histopathological image, *W *a parameter vector and *C *the still-segmentation, resulting of running a segmentation algorithm over the image *I *with parameters *W*. Provided that it is possible to define an energy function *E*(*W*, *G*, *I*) that quantifies the similarity between the still-segmentation *C *and a ground truth partition *G*, then a set of training samples *S *= {(*I^i^*, *G^i^*), *i *= 1, 2, ... *p*} corresponding to manually segmented images, will be used for finding the W* optimal vector which solves the following optimization problem:(2)

with Ω the set containing any possible parameter vector. For this problem to be solved it is necessary to define the structure of the energy function *E*(*W*, *G*, *I*).

#### Energy Function

The proposed energy function must quantify the similarity between two image partitions: the generated by the pathologist's selection and the produced by the segmentation method. This measure should cope with two different conditions: the perceptual relevance of the region center should be large and the measure should penalize miss-segmentations, i.e., classification is mainly addressed to regions rather than to pixels. We used the Mezaris metrics [39], an extension of another perceptual measure [40] which weights the visual relevance of any foreground-background segmentation:(3)

where *q_MF _*(*g*, *c*) amounts to the missing foreground pixels (MF) and *q_AB _*(*g*, *c*) to the false background pixels (AB), weighted by their distances to the closest region borders, as follows:(4)(5)

here *c *is the segmented RoI, *g *the ground truth, {·}^c ^denotes complement, *c_i _*= {**x **|**x **∈ *c*, *d*(**x**, *c*^c^) = *i*} corresponds to the set of pixels inside the RoI at the same distance from its border, *d*(**x**, *c*) is the distance between the pixel **x **and the region *c *(in this case the Euclidean distance),  = { **x **|**x **∈ *c^c^*, *d*(**x**, *c*) = *i*} stands for the set of pixels outside the RoI at the same distance from its closest border, *w_MF _*(*i*) and *w_AB _*(*i*) are the weighting functions for the missing foreground pixels and the false background pixels, both growing linearly, while *D_MF _*_max _and *D_AB _*_max _are the maximum permitted distance for the missing foreground pixels and the false background pixels, respectively.

Measure 3 was originally proposed for evaluating the segmentation quality in background-foreground segmentations, an approach which will fail in segmentations with multiple components. A multicomponent measurement was proposed by Mezaris et al. [39], exploring three error sources: inaccuracy of the region boundary location, under-segmentation and over-segmentation effects. For so doing, let

*C *= {*c*_1_, *c*_2_, ..., *c_K _*} a still-segmentation composed of regions *c_k _*and *G *= {*g*_1_, *g*_2_, ..., *g_Q_*} the ground truth partition. The inaccuracy is quantified by comparing the ground truth and the segmented images as corresponding region pairs. This correspondence is obtained by associating each ground truth region *g_q _*to the still-segmentation region *c_k _*with which the overlapped area is maximum. Once this association is established, the relationship is unique and unalterable. The inaccuracy *e_bl _*for any pair of regions is computed as follows:

where *A *is a set that contains the pairs of corresponding regions. Given that *c_k _*and *g_q _*constitute a unique couple and none of them can have a new link to another region, it is possible to obtain non coupled regions in *C *and *G*. A particular segmentation may result in a partition in which some regions have not a corresponding pair in two different situations: over and under segmentations, see Figure 4. When the actual region in the ground truth image corresponds to many regions in the segmented image, we are over-segmenting, case in which the measure penalize it by adding a term that takes into account the area defined by all these regions, as follows:

where *e_oν _*is the over-segmentation error, *A_c _*is the set of the *c_i _*non coupled regions and *B *is a black image. Similarly, when there is a non coupled region in *G *and multiple regions in the still-segmentation image *C*, the under-segmentation error is calculated as follows:

where *e_un _*is the under-segmentation error and *A_g _*is the set of the *g_i _*non coupled regions. These error sources can be combined in a single quality segmentation measure, that can be used as energy function for the learning optimization problem 2:(6)

The optimal segmentation parameters were found by a pattern search method [41], since that the energy function is not derivable.

### Automatic parameter estimation

Our model calculates multiscale "conspicuity" maps for three low level characteristics, i.e., intensity, color and orientation. These conspicuity maps are normalized and summed into the augmented conspicuities maps, whose maxima define the most relevant location. This normalization preserves information which is localized while other types of noise are ruled out. However, these low level features are not enough to conform the attention foci in the histopathological domain. The proposed model should adjust different parameters: the size of the found regions and the map weights for each of the selected features, i.e., orientation, intensity, double color opposition (*V*1 function) and entropy (*V*4 function). The best parameters were then obtained by a conventional generalized pattern search algorithm (GPS) [41], which performs better when the cost function is smooth. In the present work, that cost function corresponds to the quality measure defined in the equation 3, which is computed for any particular configuration of objects. The GPS method was thus consistently used, first for finding the optimal region size and then for setting the importance weight of each of the used features.

The GPS method constructs a sequence of iterates that converges to a stationary accumulation point. Let *k *∈ ℕ denote the iteration number, and let *x*_*k *_∈ *X *denote the current iterate. After a finite number of iterations, this algorithm searches the smaller *f *(*x*_*k*+1_), when evaluating on the points of the set *L*_*k *_= {*x *∈ *X*|*x *= *x*_*k *_± Δ_*k *_*s*_*i*_ê_*i*_, *i *∈ {1, ..., *n*}}, where Δ*_k _*> 0 is a scalar called the mesh size factor, *s_i _*∈ ℝ*^n ^*is a fixed parameter that can be used to take into account different scales and ê*_i _*is any search direction previously selected. Besides, the algorithm has a rule that selects a finite number of points in *X*, on a mesh defined by *M *(*x*_0_,Δ*_k_*) = {*x*_0 _+ *m *Δ*_k_s_i _ê_i_*|*i *∈ {1, ..., *n*}, *m *∈ ℤ}, where *x*_0 _∈ *X *is the initial iterate. If a mesh point *x' *∈ *M *(*x*_0_, Δ*_k_*) with *f *(*x'*) <*f *(*x_k_*) has been found, then the search continues with *x*_*k*+1 _= *x' *and Δ_*k*+1 _=Δ_*k*_. Otherwise, all points in *L*_*k *_are tested for a decrease in *f *(·), i.e., *f *(*x'*) ≥ *f *(*x*_*k*_) ∀ *x' *∈ *L*_*k*_, then the search continues with *x*_*k*+1 _= *x*_*k *_and a reduced mesh size factor, say Δ_*k*+1 _= Δ*_k_*/2, hence the search continues on a finer mesh. The search stops if the mesh has been refined a user-specified number of times. This algorithm evaluates then a variable neighborhood around an analysis point *p *[41].

### Assigning levels of relevance

Normal tissues appear as homogeneous architectures. Tumors and other pathologies introduce heterogeneous areas within this architecture, due to the presence of infiltrating, inflammatory and tumor cells, and the loss of marked boundaries [15]. Then, determining a measure of heterogeneity would be useful for locating the abnormal structures in the images. Heterogeneity might be understood as texture disorder that can be measured by entropy. Our approach adds the calculation of an additional conspicuity map for the intensity entropy.

Accordingly, the augmented saliency map is calculated by including intensity, color, orientation and entropy. The computed conspicuity maps, for the low level features and entropy, are considered as a process of the *V*1 area of the visual cortex and the working memory. The segmentation, provided by the aforementioned algorithm, is considered as the *V*2 visual cortex area process. This information is combined by firstly calculating an index for each low level feature from each region. This index is a pixel value average, inside the region conspicuity maps for intensity, color, orientation and entropy. Finally, the total region saliency is estimated by linearly combining and normalizing its conspicuity maps (*V*1 function) and the entropy (*V*4 function) using the learned weights (*V*1, *V*2 and *V*4 integration). The algorithm finds the relevant diagnosis structures as the most saliency ones.

## Results

### Evaluation Issues

As far as we know, this is the first investigation devoted to extract useful structural information from histopathological images, using a bio-inspired model. The developed method was compared with two well known techniques which had to deal with similar challenges, but in natural images. We used them as the base line because they also emulate the visual system, even though they are not specifically devised to detect relevancy in medical images, these two models were: 1) the Itti's model [18,29], one of the most popular algorithms to find RoIs in an image. This algorithm emulates the first 20 *ms *of the attentional process at simulating the biological model described in [42]. Basic low level retinal stimuli are non linearly weighted into a single activation map which preserves high frequency changes in a multiresolution analysis, and 2) the Achanta's model [30], a general purpose algorithm aimed to extract meaningful objects of interest. This model first computes a saliency map as the difference between the image and a blurred version, upon which the texture information is removed, obtaining a first saliency map. Salient pixels are then grouped up using a set of rules based on common locations, chrominances and luminances (Gestalt laws). Finally, relevant regions are those for which their mean is larger than the image saliency mean.

In this paper two main issues were assessed, namely the accuracy of the proposed RoI extraction method and its generalization ability (Section *Automatic Still-Segmentation of Histopathology Images*), using a total of 338 manually segmented images (Section Images and Ground Truth). Comparisons were performed between manual segmentation and the three automatic methods: Itti's, Achanta's and ours. Itti's RoIs were set at thresholding the resultant visual attention maps [43]. Likewise, the robustness of the automatic segmentation algorithm was evaluated by an 11-folding strategy, understanding this robustness as the method performance when the algorithm runs over a different set of data.

Three quality measurements were computed, the classical sensitivity and specificity and a quality segmentation measurement. The sensitivity and specificity were calculated for the whole set of classified pixels, i.e., whether or not a pixel belonged to a RoI. Classically, the performance of a method is well described using sensitivity and specificity, they account for the individual result of hits or misses. However, we are interested in finding regions of interest, i.e., collections of pixels with semantic meaning. Hence, the number of regions found by each method was also compared and the sensitivity of each method, regarding the number of RoIs, was also calculated.

### RoI extraction

**Figure 5:**
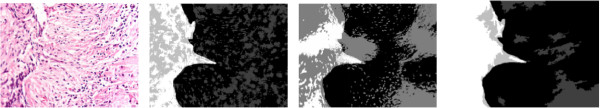
**Coincidence level between the ground truth and the evaluated models**. From left to right column: original image, the coincidence level between the ground truth and Itti's, Achanta's and our result. In the second, third and fourth columns, white and black stand for a perfect match while gray levels represent disagreement. Note that our method has much smaller scattering level and recognizes more acurately the relevant structure than the others aproaches.

Figure 5 shows a visual illustration of the differences between the ground truth segmentation and the RoI obtained using the proposed method. Coincidences between RoIs are shown in white, method misses in gray and background coincidences in black.

As observed, the proposed method is able to capture different structures of interest, in spite of the complicated patterns present in the sample. The RoI computed by our method looks perceptually more similar to the ground truth, when compared to the RoI calculated using the Itti's and Achanta's model. While the Itti's RoI looks quite scattered, our method finds a more homogeneous region, clearly much more similar to the ground truth. The Achanta's RoI completely misconfused the relevant and non relevant regions and in this case the relevant region (white region) was completely missed. In contrast, our method did find the relevant regions. Interestingly, most of misses were located near to the border, where we are supposing visual information is less important.

**Figure 6:**
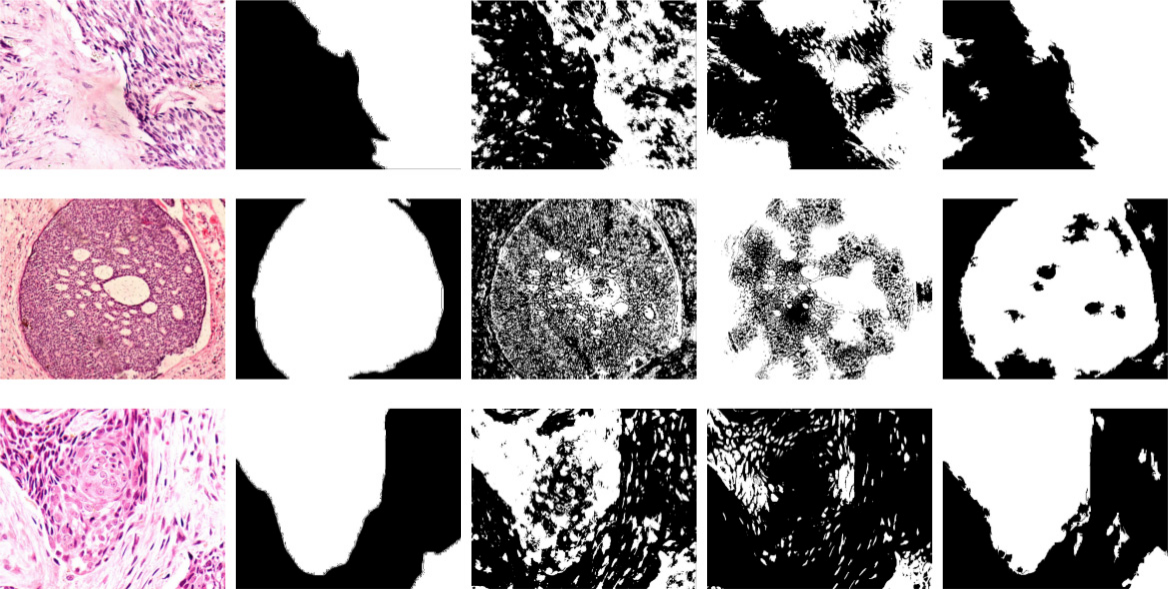
**Region found by the different methods in three random chosen images**. From left to right column: original image, ground truth, resulting RoI of the Itti's, Achanta's and our method. In the binarized images (second, third and fourth columns) the white color stands for the relevant diagnostic RoIs.

Figure 6 shows the original image in the first column, the ground truth in the second (recall white is relevant and black is not), Itti's RoIs in the third, Achanta's RoIs in the fourth and the RoIs found by our method in the fifth. The three rows show different structures, as observed in the first column. Overall, these original images show several configurations, with the carcinoma tissue in a darker violet color, which correspond to the zones highlighted in white since the expert considered them as the interest. Note that the level of structural organization is quite different so that it results impossible to determine RoIs by simply setting a set of parameters, i.e., structures present different sizes, shapes, colors and levels of hierarchy. As illustrated in the third column of Figure 6 Itti's model misses important histological objects and instead highlights many small scattered regions. This can be attributed to the fact that this model performs a pixel-based analysis and therefore it finds interesting points rather than complete defined regions. From a semantic point of view, this is a great limitation because regions with some interest are distributed all over the image, following a complex mix of rules which are in general very variable. On the other hand, the Achanta's method did not find the RoIs at all, except for the upper panel image for which it can be observed at least a well defined border between the RoI and the rest. Obviously the method was able to determine two different levels of organization at a local level, but in the mid and lower panels, the structure of interest (a node) was completely missed. A clear advantage of the proposed strategy is that nearly every spatially coherent structure was found with different levels of noise. Interestingly, most relevant objects, within these RoIs, highly coincide with what the pathologist determined as important.

### Analysis of sensitivity and specificity

The ability of the different methods to properly assign the correct label to pixels, was evaluated using conventional sensitivity and specificity measurements. These results are shown hereafter.

Table 1 shows sensitivities and specificities for each of the different magnifications and each of the assessed methods. Overall, the sensitivity measurements evidence that our method outperforms the other two at any of the different evaluated magnifications, showing a larger sensitivity for the × 20 and × 40 objective magnifications. In contrast, the three methods show comparable levels of specificity, but the larger values are shown by our method. The Itti's model presents a larger specificity at × 4, very likely because regions are not so well defined at this magnification. Recall that this evaluation was performed at the level of the pixel classification and true (false) positives (negatives) corresponded to pixels wrongly classified.

**Table 1:** Sensitivity and specifity results

*Method*	*Magnifications*	*Sensitivity (%)*	*Specificity (%)*
Itti's	× 4	63.8 ± 5.8	62.5 ± 0.3
	× 10	56.3 ± 5.8	60.3 ± 1.4
	× 20	69.7 ± 2.8	73.5 ± 0.4
	× 40	67.7 ± 3.8	73.8 ± 0.7

Achanta's	× 4	60.7 ± 8.8	62.6 ± 0.6
	× 10	53.1 ± 7.8	60.6 ± 3.1
	× 20	54.8 ± 5.8	69.6 ± 4.1
	× 40	54.8 ± 3.8	74.7 ± 5.4

Ours	× 4	**70.2 ± 13.8**	56.5 ± 6.8
	× 10	**71.2 ± 9.8**	52.1 ± 9.2
	× 20	**79.8 ± 6.8**	**77.6 ± 5.0**
	× 40	**70.7 ± 8.8**	**75.8 ± 5.0**

Sensitivity and specificity of the different evaluated methods at different microscopical objective enlargements for the whole set of correctly (wrongly) classified pixels.

The picture is completely different when one assesses the sensitivity of each method for determining correctly classified regions (rather than pixels). Table 2 presents the sensitivity of the different methods and magnifications for finding regions with meaning, in the third column, and the total number of regions whose area is smaller than a 70% of the region to which they belong and that was manually segmented by the pathologist, in the third column.

**Table 2:** Region based sensitivity.

*Method*	*Magnification*	*Sensitivity (%)*	*Number of meaningless regions*
Itti's	× 4	47.3 ± 13.7	50786
	× 10	40.0 ± 13.5	152743
	× 20	47.6 ± 17.2	23332
	× 40	44.1 ± 21.8	7879

Achanta's	× 4	45.5 ± 7.9	12207
	× 10	41.4 ± 11.3	48751
	× 20	20.3 ± 8.2	31294
	× 40	14.0 ± 8.3	7017

Ours	× 4	69.7 ± 13.6	5647
	× 10	66.0 ± 12.0	18753
	× 20	71.3 ± 14.0	1518
	× 40	61.5 ± 16.9	526

Evaluation of the different methods at several magnifications for finding regions with semantic meaning. Table shows the sensitivity in percentage and the number of regions with an area smaller than a 70% of the area marked by the pathologist to which these regions belonged.

Our method shows a sensitivity of about 70% along the four different magnifications, while the sensitivity for the other two methods ranges from 15% to 45% for the Achanta's and from about 40% to 47% for the Itti's, with comparable levels of variance. Finally, the number of regions found by our method was much more smaller, as long as the magnification is higher, and therefore with a better correlation with what the expert marked as interesting.

The next section presents the results of the Villegas-Marichal measure that evaluates the number of misclassificatons and the ability of each method to find the regions marked by the pathologist.

### Perceptual Quality Assesment

**Figure 7:**
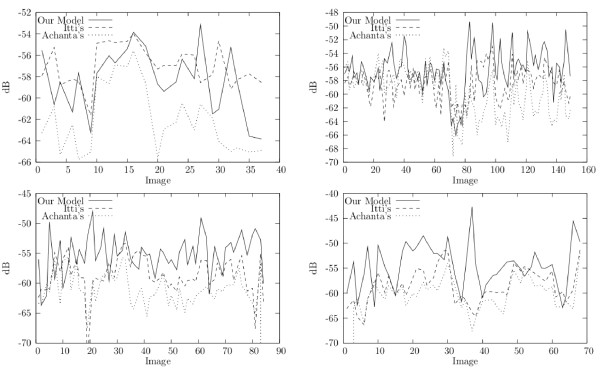
**Villegas-Marichal quality measure result by image**. The *x*-axis represents the set of available images and the *y*-axis the quality measure in decibels (*dB*).

In this section we evaluated the robustness of the proposed algorithm, that is to say, how well this strategy performs when samples change. For doing so, the set of available images was split into 11 subsets and a folding cross validation was applied for each magnification, i.e., training with 10 subsets and test with the remaining one. Figure 7 shows the performance algorithm for the whole set of available images since each image has belonged at least once to a test subset. The four panels plots the different magnifications, namely × 4 at the left upper, × 4 at the right upper, × 20 at the left lower and × 40 at the left lower panels, respectively. Each graph plots the number of available images at the *x *axis while the respective quality measurement for the three strategies (Itti's, Achanta's and ours) is plotted a the *y *axis. As expected, the RoI quality measurements vary with each image and magnification while their values range between -40*dB *and -64 *dB*. It is worthy to recall here that the more negative is this measurement the larger the number of both missing foreground and false background pixels. The graph shows a systematic gain of our method: in most images the proposed method provides better quality results. At the × 4 magnification, the Itti's model shows similar performance, likely because at this level, relevancy is associated with local color and intensity differences, while the very inner cellular structure is not yet revealed, a statement supported by the fact tha for larger magnifications (× 10, × 20 and × 40), our method clearly outperforms the others.

Overall, when applied to the test set, the proposed method outperforms Itti's at the × 10, × 20 and × 40 objective magnifications in about 2*dB*, 3.5 *dB *and 4 *dB*, and outperforms also Achanta's, for the same magnifications, in 3.4 *dB*, 5 *dB *and 5.7*dB *respectively. Again, at × 4 objective magnification the Itti's model shows a gain of about 2 *dB *over our method. In general, a gain of 1 *dB *in this kind of evaluation can be considered as a visually representative difference[40], as illustrated in Figure 6 which shows three different microscopical fields (first column) for which the pathologist has manually drawn the RoIs (second column) and the results of the three methods for each (third, fourth and fifth columns). The quality measurements for the first microscopical image and for the three evaluated methods, namely Itti's, Achanta's and ours, was -57.3 *dB*, -63.3 *dB *and -55.9 *dB*, respectively. For the second microscopical field this measurement was -59.3 *dB*, -62.2 *dB *and -55.1 *dB*, again following the same order for the methods. The evaluation measurements for the third image were -60.48 *dB*, -64.9 *dB *and -52.7 *dB*, respectively. Recall that this measurement quantifies differences between the ground truth image and the result of any of the methods. Finally, the Wilcoxon test (*p *> 0.05) was applied to the whole set of images (at every magnification) for the three evaluated methods and differences were found to be significant.

The results herein presented support this selection, the perceptual quality obtained with the proposed algorithm is around -55.3dB, an acceptable value according to the opinion of our pathologist. Likewise, table 3 shows the importance of the figure-ground segmentation mechanisms since our method outperforms Itti's, in average in 3.6 *dB *(4.9 *dB *when comparing with Achanta's). In a non linear scale, an average gain of 3.6 *dB *is an important visual improvement, as illustrated in Figure 6. Also, as shown in tables 1 and 2, our method demonstrates a better sensitivity at retrieving not only about a 70% of relevant pixels in the image, but also a similar percentage when finding entire regions, case in which our method clearly exceed Itti's and Achanta's methods in about a 20% and 30%, respectively.

**Table 3:** Perceptual quality measure results

	*Itti's model*		*Achanta's model*		*Proposed model*	
	
	*Train (dB)*	*Test (dB)*	*Train (dB)*	*Test (dB)*	*Train (dB)*	*Test (dB)*
*False fore-ground*	-57.3 ± 0.2	-56.0 ± 2.0	-60.6 ± 0.2	-59.3 ± 1.9	-52.7 ± 0.3	-51.2 ± 3.2

*Added back- ground*	-58.7 ± 0.1	-57.3 ± 1.4	-50.8 ± 0.4	-48.8 ± 5.5	-52.0 ± 0.1	-51.6 ± 1.2

*Total*	-61.8 ± 0.1	-60.3 ± 1.4	-63.0 ± 0.1	-61.6 ± 1.1	-57.7 ± 0.1	-56.7 ± 1.6

Average ± standard deviation of the perceptual quality in false foreground, added background and total quality for proposed and Itti's visual attention models obtained on the training and test sets.

## Discussion

The present article has introduced a novel strategy, a complex mix of "bottom-up" and "top-down" mechanisms, for setting RoIs in histopathological images. The model is inspired in the first phase of a pathological examination [11,20,44,45], a process largely studied which starts by scanning the slide at a low magnification zoom. So far the underlying mechanism that controls a RoI selection in histopathological samples has been poorly studied [46]. Recent studies suggest that some visual mechanisms, such as the one that allows to highlight an object from the background (figure-ground segmentation), and the visual attentional process, are connected [46]. The figure-ground segmentation models the process that occurs when an individual is exposed to a two-dimensional surface with some gentle structural differences, and then she/he separates it into parts, one of which is consciously recognized as having a distinctive form whereas the surrounding regions have not [38]. This visual segmentation mechanism follows certain invariable rules that have shown to be relevant in diagnosis of certain dermatopatologies [46]. These rules include convexity of contours, proximity of lines around it, closed contours, simple shapes, proximity and resembling among their components. The visual attention mechanism is related to the cognitive process of selectively concentrating on one aspect of the scene while ignoring others [29]. This fact suggests that the visual system is able to selectively focus on specific areas of the image, which besides are entailed with a high relevant meaning. Yet these ideas are far from being fully exploited, our approach has been able to capture these basic facts, that is to say, that relevancy is a global property somehow constructed by integrating local features. The proposed strategy is based on the interaction of the visual cortex areas *V*1, *V*2 and *V*4 [35,36], being the *V*1 cortex responsible for assigning local levels of relevance to visual inputs while the *V*2 cortex gathers together these small regions according to some weights modulated by the *V*4 cortex, which stores some learned rules: the working memory. While the *V*1 phase spans the first 20 *ms*, the others have been observed within the first 100 *ms*. This complex network of interactions ends up by selecting the relevant areas that are thus further processed in other brain areas [47]. Our model, based on this type of associations, integrates these three stages as follows: 1) Using the conventional Itti's model, a local region level of relevance is set, using exclusively low level features. This process emulates what happens in the first 20 *ms *of the visual perception 2) The saliency map coming out from the precedent phase is the input to this stage. The integration process, carried out in the first 100 *ms *is modeled by a clustering strategy, learned from the expert segmentation. Hence the low level characteristics are grouped up using an oversegmentation algorithm, which minimizes the within-class variance whose parameters are learned from the problem. 3) The saliency map is thresholded using two types of information: a measure of the texture pattern (simulates the *V*4 function) and the previouly described Itti's map (*V*1 function). The *V*4 function is herein constructed upon the base of two complementary processes, the closeness gestalt law (rough segmentation) and the grouping stratregy associated to the particular cell organization (working memory), which was estimated with a general texture measurement: the local entropy.

Many endeavours have been dedicated to segmenting areas with cancer in histopathological images. The coarse structural recognition has been already implemented as an still-segmentation algorithm, using KNN and spectral clustering [14,48], but these strategies only cope with local spatial relationships, and no perceptual meaning has been assigned. Other methods have attempted to find structures using different representations of the work of memory. Specifically, images are represented by small patches, collected together as a Bag of Features [49]. These patches are then stored in a database and used as the knowledge to which any other input must be compared. Two main issues arise with this representation: it is neither clear the number of patches used for optimally represent a concept nor the selected metrics to define similarity. Some strategies, such as the Scale Invariant Feature Transform (SIFT) or the SIFT descriptor have been used to detect the most relevant patches. However, these methods are exclusively local and very noise sensitive, crucial issues in histopathological images. Likewise, texture descriptors have been used to over-segment natural scenes, [50,51] such as we did using the luminance channel. This is very different from what we presented here because texture measurements were included after the segmentation was achieved, thereby capturing and learning from the user the configuration of local units of information, i.e., particular pixel arrangements with semantic meaning. In the present investigation we used a scalar measurement of such configurations (entropy), but notice that this measure could be replaced by more complex models, with vectorial information for instance. The advantage with this measurement is that it replaces a database and introduces the *V*4 modulation (working memory). In the present investigation two state-of-the-art visual models were used for comparison. Yet it is true that these methods were not specifically devised for medical images, they are nevertheless general purpose approaches which can be adapted to define RoIs. What we have demonstrated so far is that these general purpose visual models are not adequate for a specific domain such as the medical images, in which the prior knowledge results fundamental. As shown in Figure 6 the Itti's, model finds scattered regions that very hardly could be assembled into a unique structure, such as those drawn by the pathologist as the ground truth (Figure 6). The Achanta's method detects larger regions, but they are completely different from what the pathologist marked, very likely because it disregards texture information.

One of the most challenging issues in histopathological images regarded the fact that semantic interest is related to similarity, no matter whether these regions are neighbors or not. This drawback was herein dealt with a graph-based image segmentation algorithm [37], which in contrast to previous approaches, was capable of capturing perceptually important regions such as tissue distribution. As illustrated in Figure 6 regions obtained with the proposed strategy are perceptually more consistent and coherent with what the expert set. They are surrounded by closed contours and follow the proximity and resembling relationships, i.e., these regions satisfy the figure-ground segmentation rules. Interestingly, the ground truth also follows these figure-ground segmentation rules, as illustrated in Figure 1 a finding that supports the choice of still-segmentation methods in this type of problems. The results herein presented support this selection, the perceptual quality obtained with the proposed algorithm is around -56.7*dB*, an acceptable value according to the opinion of our expert in the domain. Likewise, table 2 shows the importance of the figure-ground segmentation mechanisms since our method outperforms Itti's, in average in 3.6 *dB *(4.9 *dB *when comparing with Achanta's). In a non linear scale, a gain of 3.6 *dB *in the mean performance is an important improvement, as illustrated in Figure 6.

## Competing interests

The authors declare that they have no competing interests.

## Authors' contributions

RG developed the algorithms and evaluated the results of the visual model. FG developed the algorithms and evaluated the results of the learning algorithm. LR constructed the ground-truth dataset. ER conceived the study, developed the fundamental ideas underlying this model, participated in the experimental design and was the director of the whole project. All authors read and approved the final manuscript.
